# Demographic Characteristics of Patients Undergoing Rhinoplasty: A Single Center Two-Time-Period Comparison

**Published:** 2017-09

**Authors:** Shirin Loghmani, Shahriar Loghmani, Hebatollah Baghi, Mohammad Ali Hoghoughi, Fariba Dalvi

**Affiliations:** 1 Department of Plastic Surgery, School of Medicine, Shahrekord University of Medical Sciences, Shahrekord, Iran; 2 Ordibehesht Surgical Center, Isfahan, Iran; 3 College of Health and Human Services, George Mason University, Fairfax, USA; 4 Department of Plastic Surgery, School of Medicine, Shiraz University of Medical Sciences, Shiraz, Iran; 5 Department of Plastic Surgery, School of Medicine, Isfahan University of Medical Sciences, Isfahan, Iran

**Keywords:** Demographic, Sex, Age, Rhinoplasty

## Abstract

**BACKGROUND:**

One of the most common cosmetic surgeries is rhinoplasty. Iran has the highest rate of rhinoplasty, worldwide. The aim of this study was to compare the demographic characteristics of patients’ undergone rhinoplasty during two-time-period with a 10-year-interval in a single surgical center in Isfahan, Iran.

**METHODS:**

In a retrospective study, data of the patients who were scheduled for elective rhinoplasty including their age and gender in two-time-period (2005 and 2015) were collected and compared.

**RESULTS:**

Data of the 470 and 472 patients’ undergone elective primary rhinoplasty during 2005 and 2015 were collected, respectively. In 2005, the age range of patients was 16-51 years. Frequency of patients aged less than 20 years and more than 40 years was 27.1% and 3%, respectively. In 2015, the age range of patients was 16-59 years. Frequency of patients aged less than 20 years and more than 40 years was 12.9% and 5.6%, respectively. Patients in the two studied periods were similar regarding gender, but the mean age of patients had a significant increase during the time.

**CONCLUSION:**

Most of our patients were female and the female to male ratio was similar in two studied periods, but it seems that rhinoplasty request is higher in older age in recent years. It is recommended to plan a trend study and more studies considering other factors to be effective in epidemiologic feature of rhinoplasty in our community.

## INTRODUCTION

Cosmetic plastic surgery has drastic popularity during the last decades, worldwide. One of the most common cosmetic surgeries is rhinoplasty.^1^^,^^2^ Evidences indicated that rhinoplasty performed more frequently among Asians.^3^ Iran has the highest rate of rhinoplasty in the world, and the rate of rhinoplasty in 2011was reported 180 cases per 100,000 populations.^4^^-^^7^ The high rate of rhinoplasty in our community may be due to factors such as low cost and improved techniques of the surgery.^8^ Further, some studies demonstrated that body dysmorphic disorders as well as other physiological problems, including depression are more prevalent among this group of patients.^9^^-^^11^

Though rhinoplasty is considered a safe procedure and most of the cases performed as elective surgery, but it is associated with many minor or major complications.^12^^,^^13^ In addition, the mentioned psychological problems mostly would continue and in some cases are deteriorated after rhinoplasty.^14^ It is suggested to properly manage the increasing trend of rhinoplasty in an acceptable range and decrease its related medical and psychological problems in our community; so we should provide a reliable and clear epidemiological feature for the procedure. This baseline information could help us in planning further interventions. The aim of this study was to represent and compare the demographic characteristics of patients’ undergone rhinoplasty during two time periods with a 10-year-interval in a single surgical center in Isfahan, Iran.

## MATERIALS AND METHODS

In a retrospective study, data were collected in 2005 and 2015 time periods from patients who referred to Ordibehesht Surgical Center, Isfahan, Iran for rhinoplasty. Data were collected regarding gender and age of the patients (n=942). Data were for the patients who were scheduled for primary rhinoplasty or septorhinoplasty. Patients with missing data and secondary rhinoplasty were excluded in this study. Demographic characteristics of the patients regarding their age and gender were compared between the two time periods of 2005 and 2015.

Obtained data were analyzed using Statistical Package for Social Sciences (SPSS, Version 20, Chicago, IL, USA). Chi-square test was applied to identify differences in responses between the two gender in the two time periods of 2005 and 2015.^15^ Two-way ANOVA was used to evaluate the year’s differences and gender differences in reference to the patients’ age. The statistical interaction between years and gender in reference to the patient’s age was assessed. Lack of interaction between years and gender demonstrated that the results obtained for gender were generalizable across years.^16^

## RESULTS

Data of the 470 and 472 patients’ undergone elective rhinoplasty during 2005 and 2015 were collected, respectively. From studied populations, 79.4% and 78% of patients in 2005 and 2015 were female, respectively. Female to male ratio was 3.58 and 3.89 in 2005 and 2015, respectively.

Demographic characteristics of the patients in the two time periods were presented in Table 1. There was no significant difference in responses between the two time periods and gender. In other words, the percentages of patients who had a surgery were similar regarding the two time periods and sex (*p*=0.32). 

**Table 1:** Demographic characteristics of patients underwent rhinoplasty in 2005 and 2015 in Ordibehesht Surgical Center in Isfahan, Iran

**Variable**	**2005** **n=470**	**2015** **n=472**	**P value**
Gender [no. (%)]			
-Female -Male	373 (79.4%) 97 (20.5%)	368 (78%) 104 (22%)	0.329
Age [mean(SD)]			
-Female -Male -Total	25.02 (6.82) 23.55 (4.63) 24.71 (6.45)	27.64 (6.76) 26.83 (6.88) 27.46 (6.79)	<0.001

There was a significant difference between the mean of patient’s age across the two time periods and gender (*p*<0.01). In 2005, the age range of patients was 16-51 years. Frequency of patients aged less than 20 years and more than 40 years was 27.1% and 3%, respectively. In 2015, the age range of patients was 16-59 years. Frequency of patients aged less than 20 years and more than 40 years was 12.9% and 5.6%, respectively. 

**Fig. 1:**
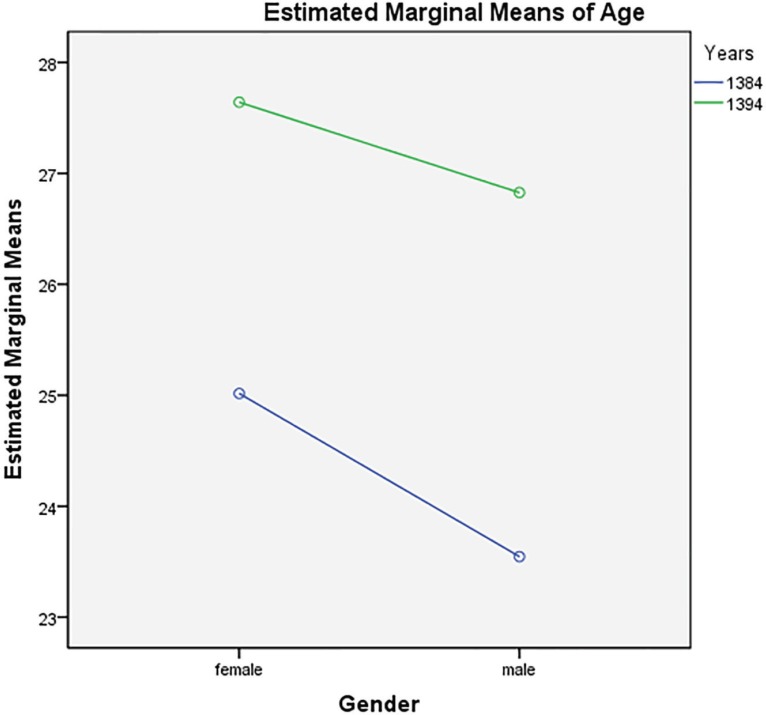
The interaction between gender and years in patients underwent rhinoplasty in 2005 and 2015

Chi-square (Chi-square=0.273, *p*=0.601) revealed that there was no significant association between gender and the two time periods of 2005 and 2015 for rhinoplasty, but there was statistically significant association between age and the two time periods of 2005 and 2015 for rhinoplasty (For gender, F=4.706, *p*=0.03, For Years, F=31.460, *p*=0.0001). No significant difference between the two time periods of 2005 and 2015 and gender (F=0.387, *p*=0.534) was noticed showing that the findings for gender and age were generalizable and stable across the two time periods of 2005 and 2015. The lack of difference between gender and the two time periods of 2005 and 2015 was displayed in Figure 1 (Estimated marginal mean of age).

## DISCUSSION

In the current study, we compared demographic characteristics of the patients’ undergone rhinoplasty during two time periods of 2005 and 2015. Our results indicated that patients in the two studied times were similar regarding gender, but the mean age of patients had a significant increase during the time. As mentioned, rhinoplasty was the most common plastic surgery in Iran and considering the findings of other researchers, it seems to have an increased trend in future.^17^ It was demonstrated that more than half of the high school girls in Kerman, Iran were interested to undergo rhinoplasty.^18^

The results of this study demonstrated that most of the patients were female and the female to male ratio was 3:4 in the two studied years. The ratio was not significantly different between studied time periods. In a study in Sweden in a population with different nationalities, the ratio was 1.3:1. They showed that the procedure was more prevalent among Middle Eastern patients including Iranians. Their results among Middle Eastern patients indicated that female to male ration was higher in younger and older patients, but in middle aged patients, the ratio was identical.^19^

In a study in Mashhad, Iran,79% of the studied population were female.^18^ Mean age of the studied population was 24 and 27 years in 2005 and 2015, respectively. The reported mean age of the two studied time periods was in the similar range that was reported in Iranian and non-Iranian populations. The median age of rhinoplasty was reported to be 26 years in Sweden and 25 years in Mashhad, Iran.^18^^,^^19^ The findings of the current study showed that the mean age of rhinoplasty increased in recent years and was higher in 2015 when compared to 2005. Frequency of patients aged less than 20 years was lower in 2015 in comparison to 2005 and frequency of patients aged more than 40 was higher in 2015 when compared to 2005, that may be due to the reason that in recent years people accepted to undergo surgery with greater awareness showing that the trend is increasing in older age population. 

However, the causes of this epidemiological feature should be investigated in future studies. Though most of the previous studies indicated that the prevalence of body dysmorphic disorders and psychological problems were higher in patients seeking rhinoplasty;^9^^-^^11^ but, in Tehran, Iran, the reports showed that the total score of mental health and self-concept of patients undergone rhinoplasty was not significantly different when compared with normal population and they recommended to evaluate different aspects of the problem including demographic, sociocultural and psychological factors too.^20^

The limitation of our study was that due to missing data, we could not record the data of the years between the two time periods of 2005 and 2015. However, recording of the data between the studied time periods would help us to determine the trend of rhinoplasty in our center, which is the main referral center in Isfahan, Iran. Totally, demographic evaluation of patients undergone rhinoplasty in two time periods in Isfahan, Iran with a 10-year interval indicated that most of the patients were female and the female to male ratio was similar in the studied time periods of 2005 and 2015, but it seems that rhinoplasty request was higher in older age in recent years. It is recommended to plan a trend study and more studies considering other factors which could be effective in epidemiologic feature of rhinoplasty in our community. 
